# Setup error assessment based on “Sphere-Mask” Optical Positioning System: Results from a multicenter study

**DOI:** 10.3389/fonc.2022.918296

**Published:** 2022-10-04

**Authors:** Yan Zhang, Han Zhou, Kaiyue Chu, Chuanfeng Wu, Yun Ge, Guoping Shan, Jundong Zhou, Jing Cai, Jianhua Jin, Weiyu Sun, Ying Chen, Xiaolin Huang

**Affiliations:** ^1^ School of Electronic Science and Engineering, Nanjing University, Nanjing, China; ^2^ Department of Radiotherapy, Nantong Tumor Hospital, Nantong, China; ^3^ Department of Radiotherapy, Suzhou Municipal Hospital, Suzhou, China; ^4^ Department of Radiation Physics, Zhejiang Cancer Hospital, Hangzhou, China

**Keywords:** S-M_OPS, CBCT, laser line, setup, radiotherapy

## Abstract

**Background:**

The setup accuracy plays an extremely important role in the local control of tumors. The purpose of this study is to verify the feasibility of "Sphere-Mask" Optical Positioning System (S-M_OPS) for fast and accurate setup.

**Methods:**

From 2016 to 2021, we used S-M_OPS to supervise 15441 fractions in 1981patients (with the cancer in intracalvarium, nasopharynx, esophagus, lung, liver, abdomen or cervix) undergoing intensity-modulated radiation therapy (IMRT), and recorded the data such as registration time and mask deformation. Then, we used S-M_OPS, laser line and cone beam computed tomography (CBCT) for co-setup in 277 fractions, and recorded laser line-guided setup errors and S-M_OPS-guided setup errors with CBCT-guided setup result as the standard.

**Results:**

S-M_OPS supervision results: The average time for laser line-guided setup was 31.75s. 12.8% of the reference points had an average deviation of more than 2 mm and 5.2% of the reference points had an average deviation of more than 3 mm. Co-setup results: The average time for S-M_OPS-guided setup was 7.47s, and average time for CBCT-guided setup was 228.84s (including time for CBCT scan and manual verification). In the LAT (left/right), VRT (superior/inferior) and LNG (anterior/posterior) directions, laser line-guided setup errors (mean±SD) were -0.21±3.13mm, 1.02±2.76mm and 2.22±4.26mm respectively; the 95% confidence intervals (95% CIs) of laser line-guided setup errors were -6.35 to 5.93mm, -4.39 to 6.43mm and -6.14 to 10.58mm respectively; S-M_OPS-guided setup errors were 0.12±1.91mm, 1.02±1.81mm and -0.10±2.25mm respectively; the 95% CIs of S-M_OPS-guided setup errors were -3.86 to 3.62mm, -2.53 to 4.57mm and -4.51 to 4.31mm respectively.

**Conclusion:**

S-M_OPS can greatly improve setup accuracy and stability compared with laser line-guided setup. Furthermore, S-M_OPS can provide comparable setup accuracy to CBCT in less setup time.

## 1 Introduction

Radiotherapy is an important treatment for cancer, which can be used alone or in combination with chemotherapy and surgery to improve patient survival or prolong life (1–3). And the accuracy of radiotherapy setup directly determines the effect of fractional treatment (2, 4). Nowadays, the most frequently setup method is using thermoplastic combine markers for the patient positioning in fractions, and re-acquiring images for the positioning verification when necessary (5, 6). Commonly used image acquisition technologies for setup include cone beam computed tomography (CBCT), electronic portal imaging device (EPID), magnetic resonance imaging (MRI), binocular X-ray image guidance (including implanted gold fiducial markers), etc. (5, 7). Among them, CBCT has become the most important imaging tool for the radiotherapy setup in the past several years considering its unique advantages: three-dimensional imaging, sufficient contrast and low radiation dose, etc. (8, 9). Recent studies have shown that acquiring image for positioning verification in each fraction is beneficial to improve positioning accuracy. But these image acquisition technologies not only make patients suffer from additional radiation, but also cause extra time consumption, burdening those countries and regions with insufficient radiotherapy resources (5, 10, 11). Taking China as an example, there were 4.57 million new cancer cases in 2020, accounting for 23% of the global new cancer cases (19.29 million cases) (12, 13). However, the rate of radiotherapy equipment per million population in China was only 1.5 (14), which was lower than the WHO requirement (4 devices per million population) (15). Furthermore, other low- and middle-income countries (LIMICs) have more scant radiotherapy resources (16). Therefore, considering the time consumption and additional radiation dose, the number of unnecessary image-guided setup should be generally minimized (17–19).

In view of the above problems, many new setup methods have been proposed, including Catalyst (20), Sentinel (21), ExacTrac (7, 22–24), etc. Catalyst and Sentinel use structured light to capture 3D surface of the patient, and register the acquired surface to the previous recorded one for setup error detection. ExacTrac is assisted by two orthogonal KV-level X-ray imaging systems. Although above methods adopt new technologies in clinic, the improvement of the speed, accuracy and stability is limited. In addition, for some daily setup methods, represented by the laser line, it is also difficult to achieve high-precision and high-stability tumor positioning.

In this study, in order to assess the setup speed, accuracy and stability of "Sphere-Mask" Optical Positioning System (S-M_OPS), we used S-M_OPS to collect clinical setup data, and used co-setup experiment of S-M_OPS, laser line and CBCT to verify the feasibility of S-M_OPS for fast and accurate setup.

## 2 Materials and methods

### 2.1 S-M_OPS

S-M_OPS is an infrared optical positioning system that enables non-invasive precise positioning during setup and real-time tracking during treatment (25). It adopts rigid registration, which has been proven reliable (26). S-M_OPS consists of the infrared binocular camera, thermoplastic mask, positioning spheres, and S-M_OPS treatment planning system (S-M_OPS TPS), which can provide functions such as calculation, registration, monitoring, recording and early warning.

**Figure 1:**
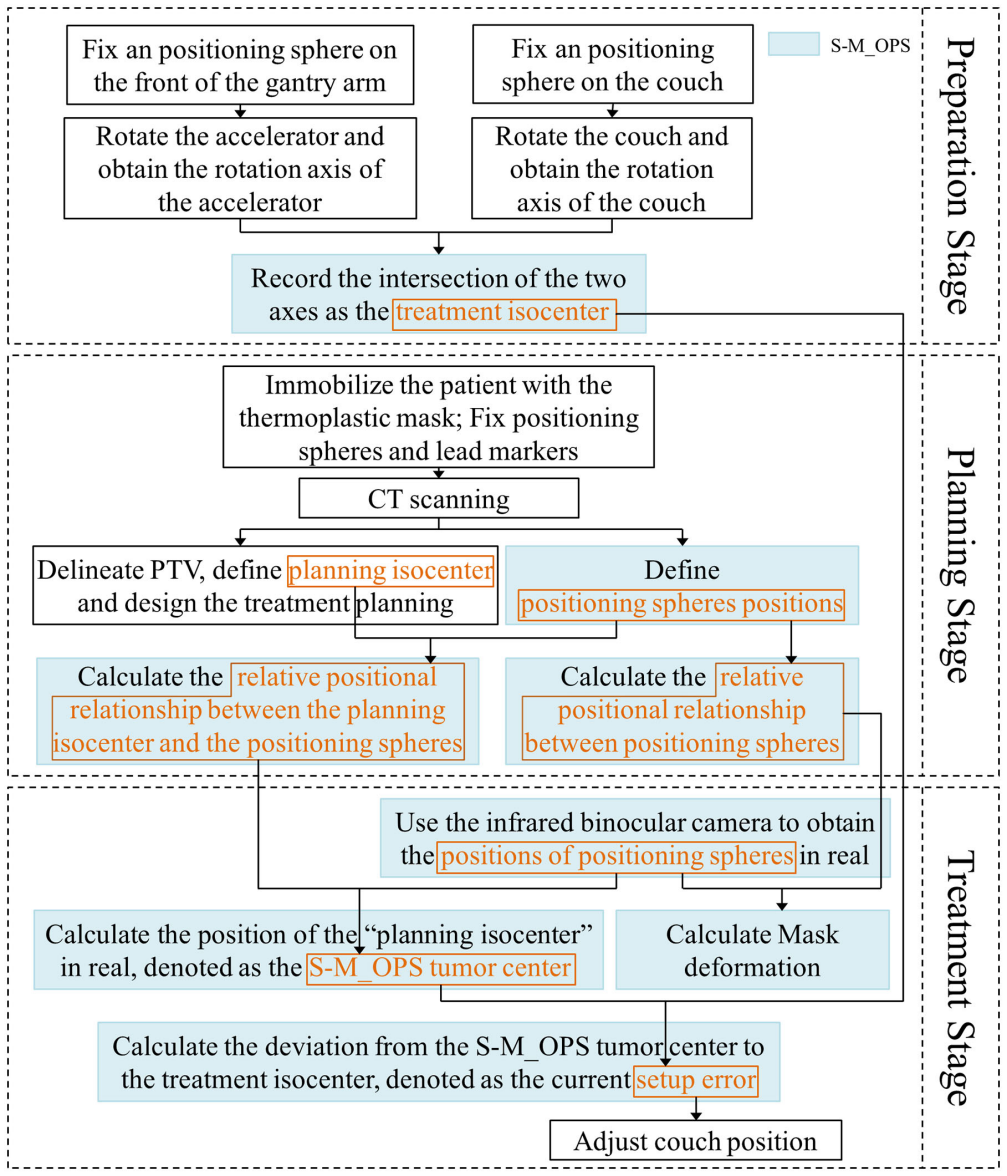
The workflow of S-M_OPS.

Process of S-M_OPS can be mainly divided into preparation stage, planning stage and treatment stage, as shown in **Figure 1**:

Preparation stage: The purpose is to record the position of treatment isocenter. The detailed procedures are listed as follows: 1) Through the stickum on the bottom of the spheres, fix an infrared positioning sphere on the front of the gantry arm and on the couch close to the linear accelerator, respectively; 2) Rotate the accelerator and the couch; 3) Track the positioning spheres by infrared binocular camera, and obtain the rotation axes of the accelerator and the couch; 4) Record the intersection of the two axes (or the midpoint of the common perpendicular line of the two axes) as the treatment isocenter. Note: Considering that the relative position between infrared binocular camera and couch is fixed, it is sufficient to register once. However, in order to reduce the influence of mechanical error on the setup accuracy, the isocenter position should be obtained once a day and the registration repeatability error of treatment isocenter should be kept within 0.5mm (If repeatability error is greater than 0.5mm, arrange for the staff to calibrate the accelerator).

**Figure 2:**
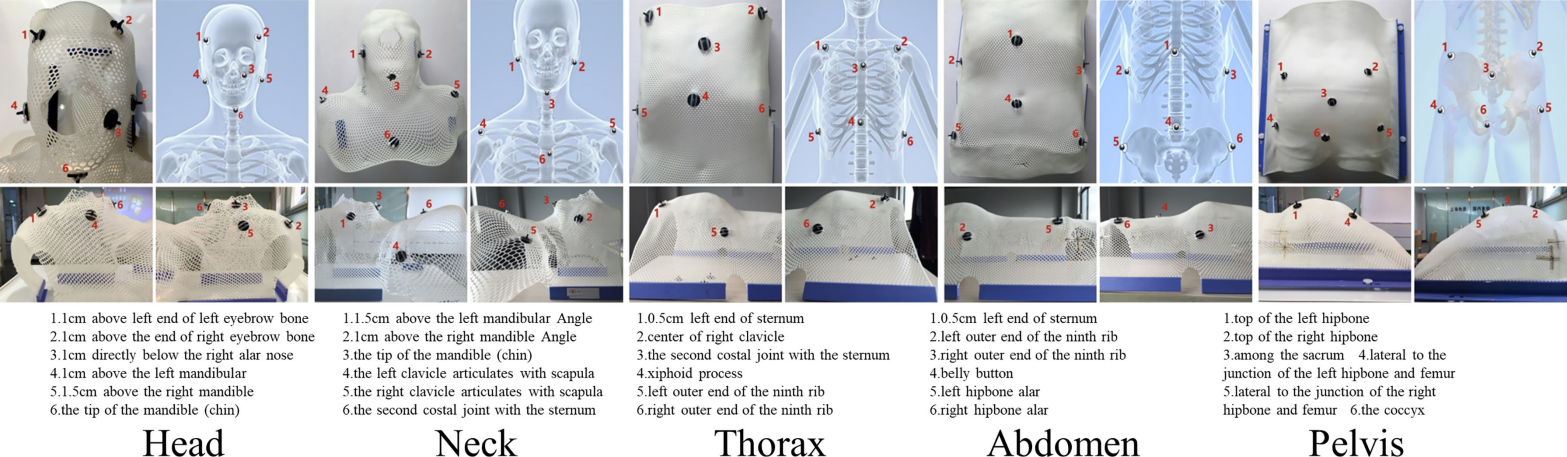
Reference positions of the positioning spheres in different parts (Upper left: top view; Upper right: skeletal diagram; Lower left: left view; Lower right: right view).

Planning stage: The purpose is to acquire the position of planning isocenter, the relative positional relationship between positioning spheres, and relative positional relationship between the planning isocenter and the positioning spheres. The detailed procedures are listed as follows: 1) Immobilize the patient with the thermoplastic mask, and fix the positioning spheres on the thermoplastic mask permanently according to the standard sphere positions (as shown in **Figure 2**, the positioning spheres are located at the bony markers). 2) Perform the CT scan. 3) The physicist delineates PTV, defines planning isocenter (the intersection of the lead marks) and designs the treatment plan. 4) S-M_OPS TPS loads CT images and recognizes positioning spheres positions in the CT images. 5) S-M_OPS TPS loads the treatment plan and obtains the planning isocenter. 6) S-M_OPS TPS calculates the relative positional relationship between positioning spheres according to the positions of positioning spheres in the CT image. 7) S-M_OPS TPS calculates the relative positional relationship between the planning isocenter and the positioning spheres according to the positions of positioning spheres and the position of the planning isocenter.

Treatment stage: The purpose is to calculate the setup error. The detailed procedures are listed as follows: 1) S-M_OPS uses infrared binocular camera to obtain the positions of positioning spheres in real. 2) According to positions of positioning spheres and the relative positional relationship between the planning isocenter and the positioning spheres (obtained in the planning stage), S-M_OPS TPS calculates the position of the “planning isocenter” in real, denoted as the S-M_OPS tumor center. 3) According to the treatment isocenter obtained in the preparation stage, S-M_OPS TPS calculates the deviation from the S-M_OPS tumor center to the treatment isocenter, denotes as the current setup error. 4) The radiotherapist can move the couch according to the setup error calculated by S-M_OPS TPS. 5) Meanwhile, S-M_OPS TPS can calculate the mask deformation according to the change of the relative positional relationship between positioning spheres.

### 2.2 Supervision setting and population

We cooperated with 12 hospitals in China (Appendix p1) from 2016 to 2021. Use S-M_OPS to supervise 15441 fractions in 1981 patients (with cancers in intracalvarium, nasopharynx, esophagus, lung, liver, abdomen or cervix) undergoing intensity-modulated radiation therapy (IMRT), and to record the data such as registration time, mask deformation and setup error.

Note: Registration time referred to the time for aligning laser lines to crosshairs on the thermoplastic mask. Mask deformation was defined as the geometric distance *L_i_ (* $L_{i}=\sqrt{{\left(A_{i}-B_{i}\right)}^{2}}$ ) between the sphere *i*’s position *B_i_* during the treatment stage and the sphere *i*’s position *A_i_* during the planning stage (*i = 1…N*, *N* was the number of spheres supervised). The setup error referred to the laser line-guided error calculated with CBCT-guided setup result as the standard.

### 2.3 Co-setup setting and population

**Figure 3:**
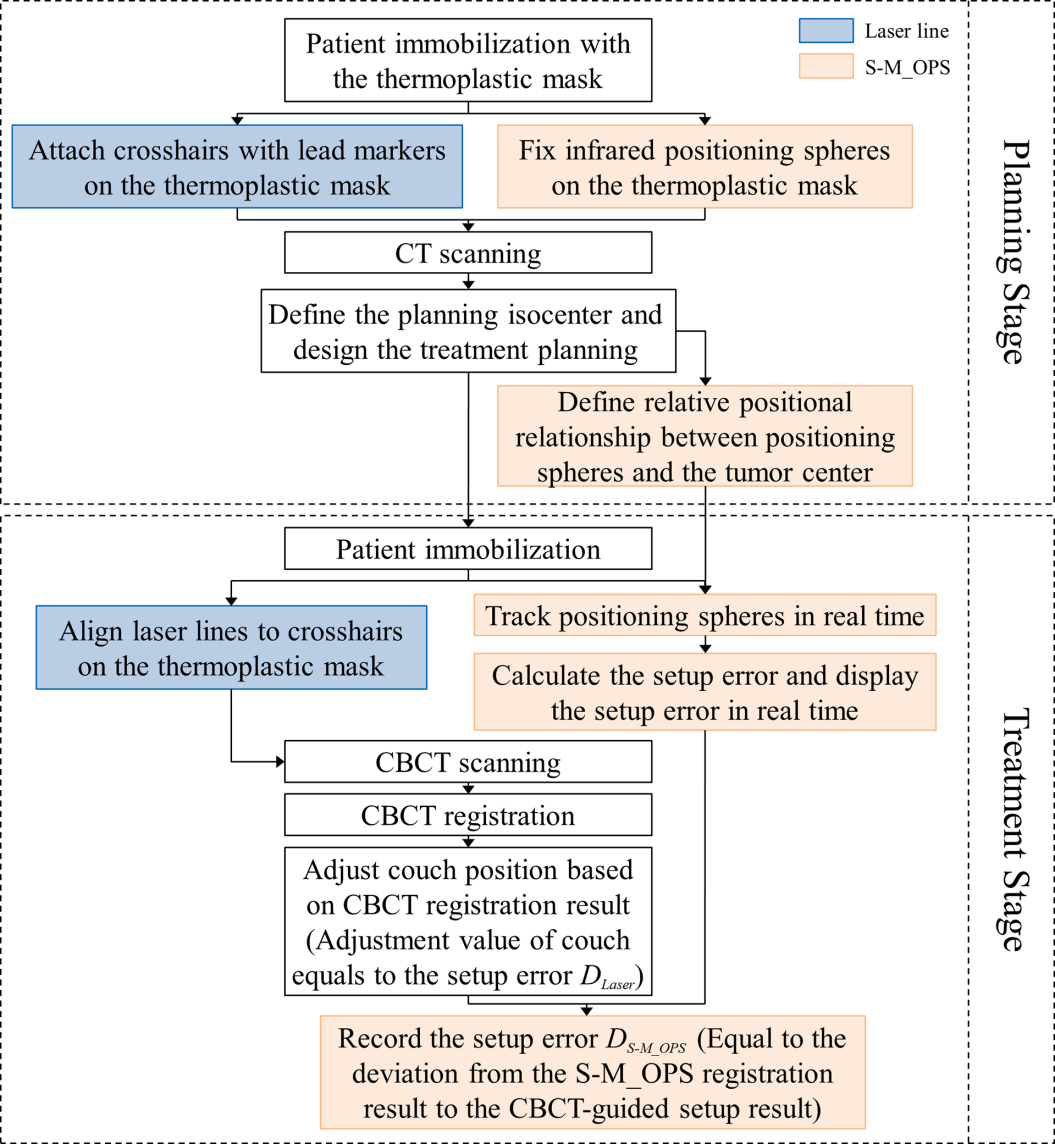
The workflow of clinical co-setup experiment.

We randomly selected 277 from 15441 fractions mentioned above for the co-setup of S-M_OPS, laser line and CBCT. The specific clinical experiment flow is shown in **Figure 3**. In addition, we performed the preparation stage of S-M_OPS before each fraction, and confirmed the repeatability error was less than 0.5mm.

**Figure 4:**
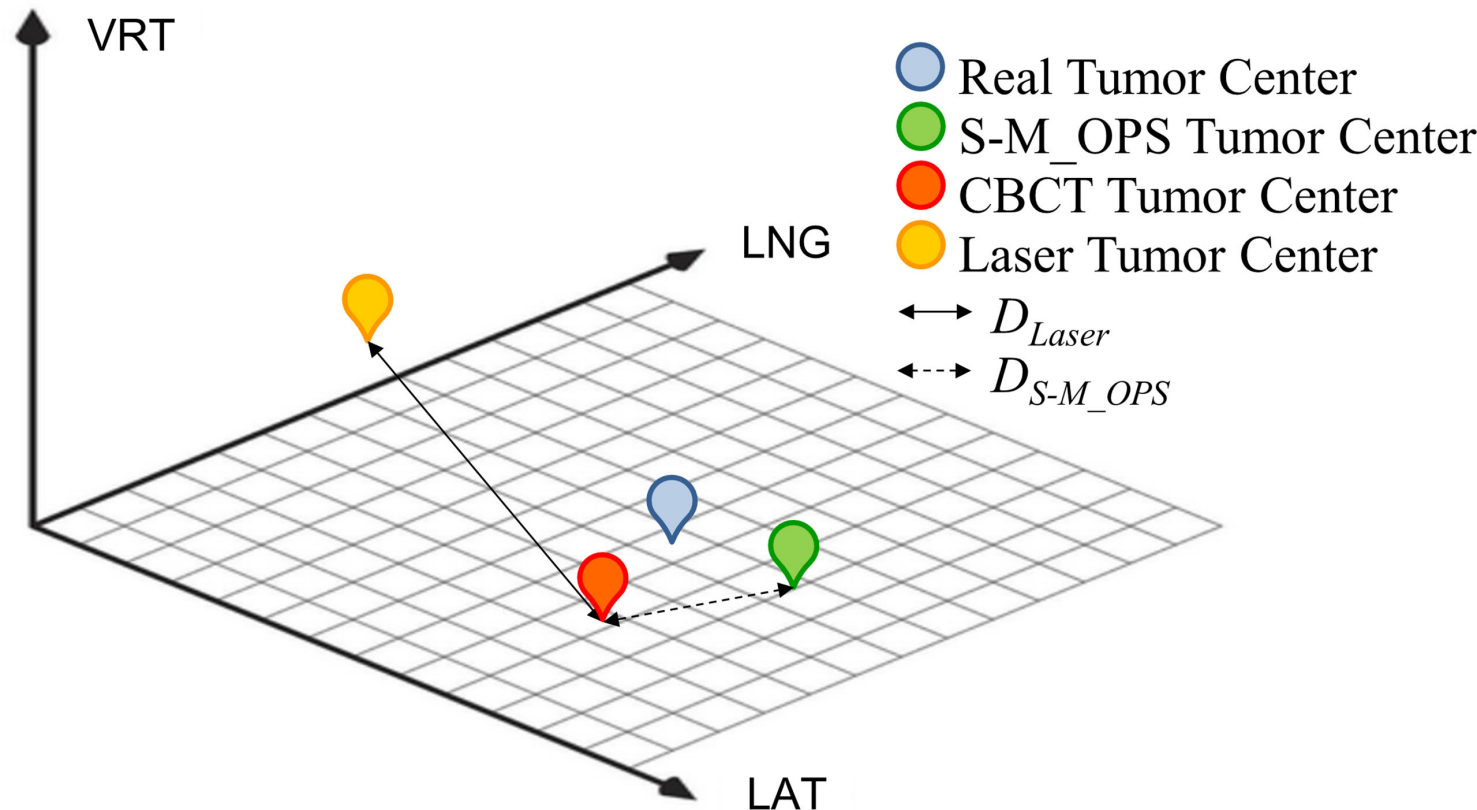
Schematic diagram of CBCT tumor center, laser line tumor center, S-M_OPS tumor center, *D_Laser_* and *D_S-M_OPS_*.

In this study, we arranged three physicists with more than 5 years of work experience for manual verification of CBCT automatic registration results, and took the average of the manual verification results as final CBCT registration result. As shown in **Figure 4**, the CBCT tumor center location (denoted as CBCT tumor center) was obtained from CBCT registration, the laser line tumor center location (denoted as laser line tumor center) was obtained from laser line registration and the S-M_OPS tumor center location (denoted as S-M_OPS tumor center) was obtained from S-M_OPS registration. Taking CBCT registration result as reference, we calculated the laser line-guided setup error (denoted as *D_Laser_*, as shown in **Figure 4**) and the S-M_OPS-guided setup error (denoted as *D_S-M_OPS_*, as shown in **Figure 4**). *D_Laser_* was defined as the deviation from the laser line tumor center to the CBCT tumor center, and *D_S-M_OPS_* was defined as the deviation from the S-M_OPS tumor center to the CBCT tumor center.

For laser line-guided setup errors and S-M_OPS-guided setup errors, *F*-test was adopted to test for equality of variances. Statistical significance was defined as *p*<0.05.Note: In the co-setup study, patients were scanned with the Brilliance^TM^ Big Bore CT Scanner (PHILIPS, Eindhoven, Netherlands), with 3mm slice thickness for head and 5mm for chest and abdomen. The treatment planning system was Pinnacle treatment planning system (PHILIPS, Eindhoven, Netherlands) and the CBCT was XVI system (Elekta, Stockholm, Sweden).

This study was approved by the hospital ethics committee. All patients involved provided written informed consent before participating into the study.

## 3 Results

### 3.1 S-M_OPS supervision results

1981 patients consisted of 1223 males (61.7%) and 758 females (38.3%), with average age of 63.6 years and a median age of 66 years. We finally obtained 4949 valid data of registration time, 15441 valid sets of mask deformation (a total of 78443 valid reference points) and 13827 valid sets of setup errors.

**Figure 5:**
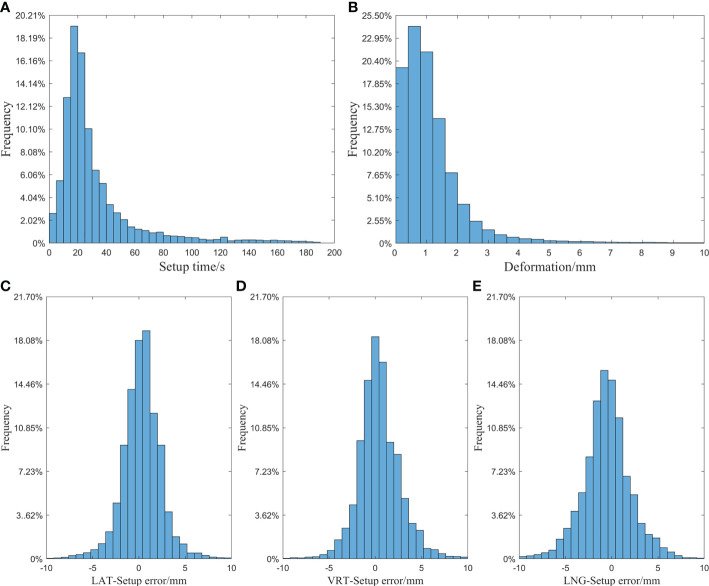
**(A)** Histogram of setup time of laser line; **(B)** Histogram of mask deformation; **(C)** Histogram of setup error in the LAT direction; **(D)** Histogram of setup error in the VRT direction; **(E)** Histogram of setup error in the LNG direction.

Registration time: The average time was 31.75s, the standard deviation (SD) was 29.42s, and the median was 22s. The specific distribution is shown in **Figure 5A**.

Mask deformation: The data distribution is shown in **Figure 5B**: the average deformation was 1.14±1.16mm, and the median was 0.90mm. Among all the reference points, 87.2% were deformed by less than 2mm, and 94.8% were deformed by less than 3mm.

Setup error: Taking the S-M_OPS registration result as the standard, the laser line-guided setup error distributions in different directions were shown in **Figures 5C–E**. In the LAT, VRT and LNG directions, the 95% CIs of the setup errors were -3.75 to 4.42mm, -3.92 to 4.84mm and -5.50 to 4.51mm respectively, and the setup errors (mean ± SD) were 0.34±2.09mm, 0.46±2.24mm and -0.49±2.55mm respectively.

### 3.2 Co-setup results

#### 3.2.1 *D_Laser_* and *D_S-M_OPS_*

**Figure 6:**
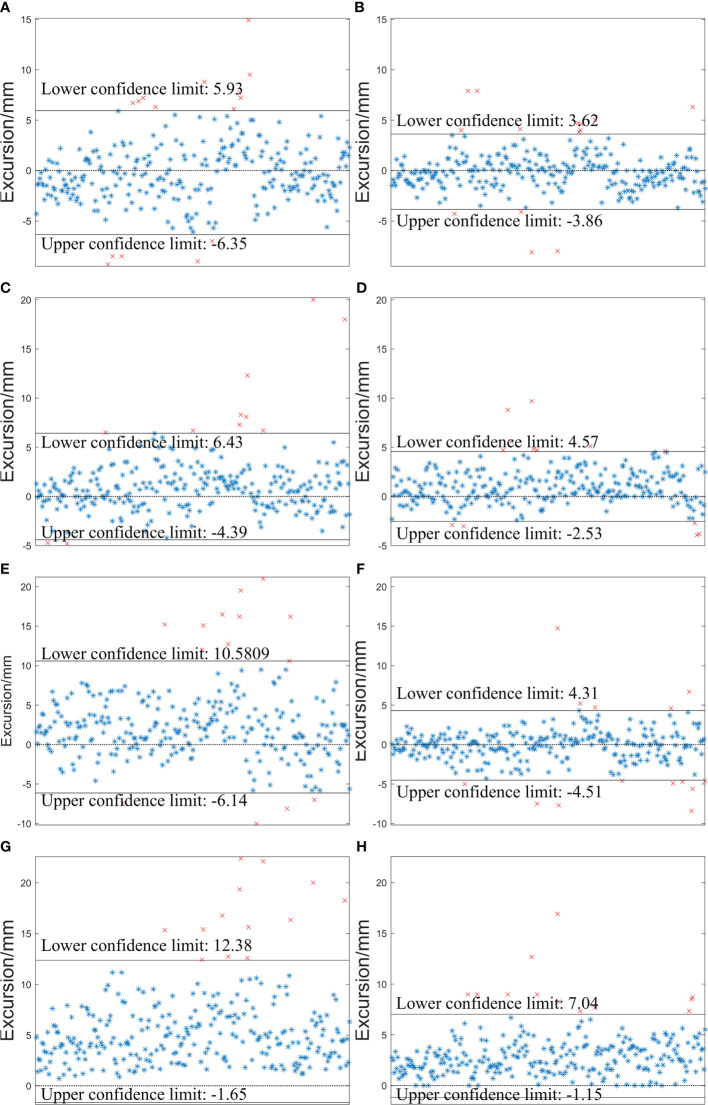
Distributions of *D_Laser_* and *D_S-M_OPS_* in the LAT (left/right), VRT (superior/inferior) and LNG (anterior/posterior) and *D (* $\text{D=}\sqrt{{\text{LAT}}^{2}+{\text{LNG}}^{2}+{\text{VRT}}^{2}}$ *)* directions. **(A)** *D_Laser_* -LAT; **(B)** *D_S-M_OPS_* -LAT; **(C)** *D_Laser_* -VRT; **(D)** *D_S-M_OPS_* -VRT ; **(E)** *D_Laser_* -LNG; **(F)** *D_S-M_OPS_* -LNG; **(G)** *D_Laser_* -D; **(H)** *D_S-M_OPS_* -D.

The specific distributions of *D_Laser_* and *D_S-M_OPS_* are shown in **Figure 6**. In LAT (left/right), VRT (superior/inferior), LNG (anterior/posterior) and D ( $\text{D=}\sqrt{{\text{LAT}}^{2}+{\text{LNG}}^{2}+{\text{VRT}}^{2}}$ ) directions, the setup errors (mean±SD) of *D_Laser_* were -0.21±3.13mm, 1.02±2.76mm, 2.22±4.26mm and 5.36±3.58mm respectively, and the 95% confidence intervals (95% CIs) of *D_Laser_* were -6.35 to 5.93mm, -4.39 to 6.43mm, -6.14 to 10.58mm and -1.65 to 12.38mm respectively. The setup errors (mean±SD) of *D_S-M_OPS_* were 0.12±1.91mm, 1.02±1.81mm, -0.10±2.25mm and 2.94±2.09mm respectively, and the 95%CIs of *D_S-M_OPS_* were -3.86 to 3.62mm, -2.53 to 4.57mm, -4.51 to 4.31mm and -1.15 to 7.04mm respectively. It indicated that S-M_OPS-guided setup accuracy and stability were better than those of laser line-guided in all directions. The results of *F*-test were showed in **Table 1**. A significant difference favouring S-M_OPS in all direction was observed (*p*<0.01 in all direction).

**Table 1:** Comparisons among laser line-guided setup errors and S-M_OPS-guided setup errors.

		LAT		VRT		LNG		D	
		Mean ± SD	*p*	Mean ± SD	*p*	Mean ± SD	*p*	Mean ± SD	*p*
All	Laser	-0.21 ± 3.13	<10^-15^	1.02 ± 2.76	<10^-11^	2.22 ± 4.26	<10^-24^	5.36 ± 3.58	<10^-17^
	S-M_OPS	0.12 ± 1.91		1.02 ± 1.81		-0.10 ± 2.25		2.94 ± 2.09	
Intracalvarium	Laser	-1.24 ± 1.52	0.01	-0.22 ± 1.52	0.21	2.00 ± 2.56	<10^-4^	3.67 ± 1.72	<10^-3^
	S-M_OPS	-0.66 ± 0.94		0.32 ± 1.29		-0.03 ± 1.09		1.85 ± 0.84	
Nasopharynx	Laser	-1.20 ± 1.40	0.02	0.13 ± 1.91	0.03	2.09 ± 3.00	<10^-6^	3.92 ± 2.15	<10^-2^
	S-M_OPS	-0.64 ± 0.88		1.25 ± 1.26		-0.06 ± 0.89		1.93 ± 1.15	
Esophagus	Laser	-0.30 ± 3.29	<10^-2^	1.05 ± 2.36	0.29	2.18 ± 3.46	<10^-8^	5.10 ± 2.83	<10^-2^
	S-M_OPS	0.23 ± 2.32		0.95 ± 2.22		-0.54 ± 1.78		3.15 ± 2.17	
Lung	Laser	-1.65 ± 3.83	<10^-5^	1.39 ± 2.21	0.06	2.91 ± 3.34	0.44	5.84 ± 3.02	0.45
	S-M_OPS	-0.40 ± 1.86		1.21 ± 1.68		0.03 ± 3.43		3.15 ± 3.08	
Liver	Laser	2.79 ± 2.10	0.06	2.02 ± 1.91	0.05	6.43 ± 5.15	<10^-7^	7.76 ± 5.22	<10^-4^
	S-M_OPS	1.87 ± 1.51		1.67 ± 1.37		2.04 ± 1.55		3.54 ± 2.09	
Abdomen	Laser	-0.28 ± 2.52	0.12	1.40 ± 3.98	<10^-3^	0.82 ± 3.28	0.43	4.93 ± 3.25	0.02
	S-M_OPS	-0.29 ± 2.01		-0.04 ± 2.01		-0.26 ± 3.17		3.64 ± 2.14	
Cervix	Laser	-0.25 ± 3.16	<10^-10^	0.98 ± 3.49	<10^-10^	1.00 ± 5.38	<10^-13^	5.79 ± 4.37	<10^-13^
	S-M_OPS	-0.73 ± 1.35		1.39 ± 1.45		-0.41 ± 1.94		2.78 ± 1.58	

Values are shown in mean ± SD and *p*-value (*F*-test).

#### 3.2.2 *D_Laser_* and *D_S-M_OPS_* of different parts

**Figure 7:**
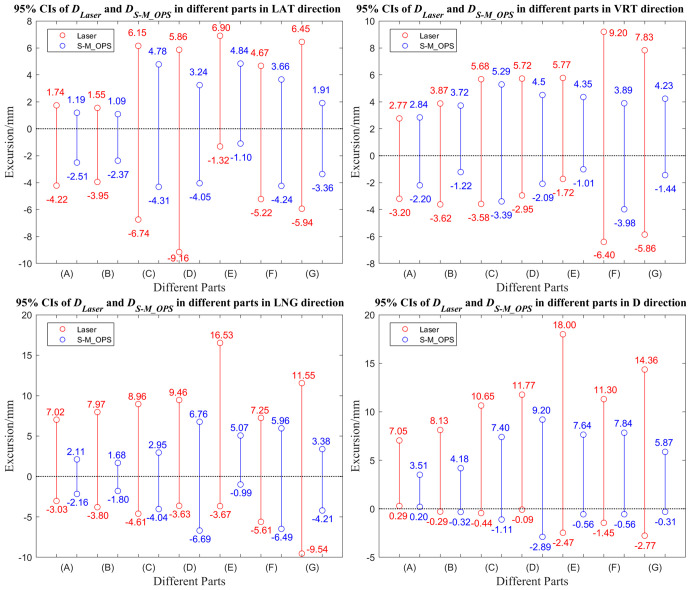
95% Confidence intervals of *D_Laser_* and *D_S-M_OPS_* in different parts in the LAT, VRT, LNG and D directions. **(A)** Intracalvarium; **(B)** Nasopharynx; **(C)** Esophagus; **(D)** Lung; **(E)** Liver **(F)** Abdomen; **(G)** Cervix.

We subdivided 277 sets of data into cancer in intracalvarium (24 cases), nasopharynx (21 cases), esophagus (80 cases), lung (35 cases), liver (25 cases), abdomen (28 cases) and cervix (64 cases). The mean±SD of *D_Laser_* and *D_S-M_OPS_* of above parts in the LAT, VRT, LNG and D directions are shown in **Table 1**, and the 95% CIs are shown in **Figure 7**.

### 3.3 Clinical setup consistency

**Figure 8:**
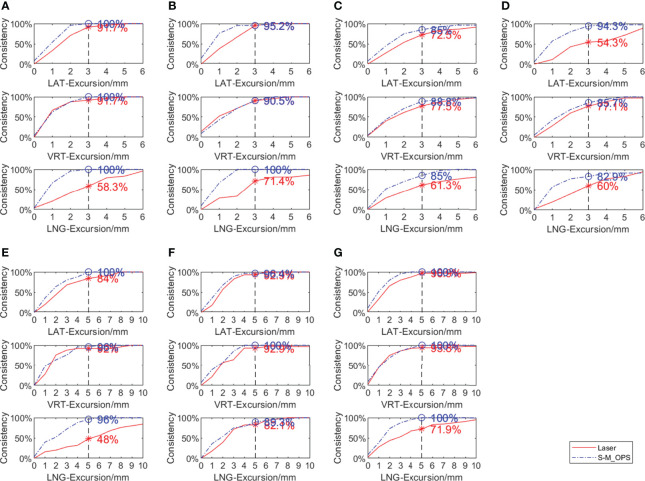
Comparison between laser line-guide setup consistency and S-M_OPS-guide setup consistency in different parts in the LAT, VRT, and LNG directions **(A)** Intracalvarium; **(B)** Nasopharynx; **(C)** Esophagus; **(D)** Lung; **(E)** Liver **(F)** Abdomen; **(G)** Cervix.

In addition to the SD and 95% CI, clinical setup consistency is also a crucial index of the setup stability. The clinical setup consistency can be defined as the proportion of the setup error meets the setup requirements clinically. For different parts, the clinical setup consistency has different requirements. For head, neck and thorax, setup error less than ±3.0 mm can be considered to meet clinical setup requirement, and for abdomen and cervix, setup error shouldn’t be greater than ±5.0 mm (27–29). **Figure 8** shows the laser line-guided setup consistencies and S-M_OPS-guided setup consistencies in different parts and directions. It showed that S-M_OPS could better meet the clinical setup requirements on various parts in all directions.

## 4 Discussion

We can evaluate a setup method from multiple perspectives generally, such as setup accuracy, setup stability, setup time and safety. High setup accuracy and setup stability can reduce additional radiation and improve safety. The shorter setup time can not only reduce patients discomfort caused by prolonged immobility, but also greatly improve the utilization efficiency of radiotherapy equipment, which is especially important for countries with insufficient radiotherapy resources. From the supervised results, the mean time for laser line-guided setup was 31.75s. In addition, through our records, the mean time for CBCT-guided setup was 228.84s (including the time for CBCT scan and the time for manual verification of automatic registration results). The mean time for S-M_OPS-guided setup was 7.47s. We find compared with CBCT-guided setup, S-M_OPS-guided setup does not require time for imaging and manual verification, which reduces setup time significantly. What's more, the CBCT-guided registration result or the laser line-guided registration result is physicist-dependent, which can be affected by personal experience. In contrast, S-M_OPS adopts point-optimized registration algorithm. It can provide unique registration result based on mathematical optimization calculations.

In addition to setup time, the mask deformation is also often overlooked by radiotherapists. It is generally caused by patient’s wrong posture, inaccurate setup, respiratory movement and body size change. The data (**Figure 5B**) showed that 12.8% of reference points had an average deviation of more than 2 mm and 5.2% had an average deviation of more than 3 mm in fractions. Therefore, if the setup error is small but the mask deformation is large, it may result from the patient's wrong posture. So the patient should be re-immobilized. What’s more, after a long treatment cycle, there may exist some changes of the patient's body size. Under this circumstance, the thermoplastic mask should be reshaped and the radiotherapy treatment plan should be remade, especially for obese patients.

Throughout the co-setup experiment, in order to reduce the uncertainties caused by mechanical error and human factor, we performed the preparation stage of S-M_OPS first to reduce the uncertainty caused by linear accelerator. In addition, we arranged three experienced physicists for manual verification in order to exclude the influence of human factors. However, we took the mechanical error of laser line and the human factor of laser line-guided registration into consideration, because these uncertainties were unavoidable for laser line-guided setup.

First, according to **Table 1**, it showed that S-M_OPS provided better setup accuracy than laser line, especially in the LNG direction. This was mainly due to the presence of slice thicknesses of 2 to 5 mm in CT data. In the radiotherapy planning stage, the lead marks were fixed on the thermoplastic mask, and lead marks were imaged by CT. The intersection of lead marks in the CT image was the planning isocenter, and the positions of lead markers on the thermoplastic mask were the positions where laser lines were aligned. Ideally, the intersection of the laser lines should be the location of the planning isocenter for radiotherapy. However, due to the thickness of CT slices, the lead markers appeared in multiple consecutive slices, and the center of the lead marker was not necessarily imaged on a specific layer. So this will lead to a deviation from the selected planning isocenter to the ideal planning isocenter. And it would lead to the deviation from the selected planning center to the intersection of laser lines (ideal planning isocenter), especially in LNG direction. While S-M_OPS uses the positioning spheres with diameter of 11mm, which can be imaged by the CT and show up in at least 2 slices. According to different cross-sections’ diameters of the same positioning sphere on consecutive CT slices, S-M_OPS can accurately calculate the position of positioning sphere’s center in combination with the geometric relationship. Therefore, there is no necessity to image S-M_OPS positioning sphere center on a specific CT slice, which makes S-M_OPS provide high setup accuracy in the LNG direction. And it can also qualitatively draw the above conclusion from the supervision results of S-M_OPS (mean, SD and 95% CI of **Figures 5C, D, E**), which shows laser line-guided setup results and S-M_OPS-guided setup results have the largest difference in the LNG direction.

Second, according to the shorter confidence intervals (**Figures 6**, **7**) and smaller SDs (**Table 1**), it also concluded that S-M_OPS could provide higher setup stability (*p*< 0.01 in all directions). High setup stability was attributed to the fact that S-M_OPS used multiple reference markers (6 positioning spheres) and selected 3 to 6 markers for registration. These selected marks were most consistent with the relative positional relationship between positioning spheres obtained during the treatment planning stage. So S-M_OPS is more likely to reduce setup errors and provides the higher setup stability.

Compared with the laser line, S-M_OPS significantly improved the clinical setup consistency in all parts. Especially in the areas with many bony structures such as the intracalvarium and pelvis, the setup consistencies of S-M_OPS all reached 100% in LAT, VRT and LNG directions. In addition, our experiments also showed that the S-M_OPS-guided setup consistencies were not prominent when applied in the esophagus and lungs, where S-M_OPS-guided setup consistencies were less than 90% in most directions. It was mainly due to the influence of respiratory movement, and deformation of thermoplastic mask was difficult to reflect the position changes of organs and tissue accurately. However, it might also be caused by the following reason: this study was based on CBCT-guided setup results, and CBCT was not suitable for monitoring intra-fraction motion considering the time spent on scan and reconstruction. Therefore, it remains to be further studied whether the S-M_OPS-guided setup consistency can be calculated based on CBCT-guided setup result in the esophagus and lung.

And finally, compared with the Sentinel, Catalyst and ExacTrac, S-M_OPS-guided setup errors were smaller than those of Sentinel and Catalyst in all parts and directions. And S-M_OPS-guided setup errors were smaller than those of ExacTrac in the vast majority of the setup results (shown in **Table 2**) (20–22, 30, 31). For Sentinel and Catalyst, the setup accuracy is mainly affected by the following three factors. First, there is not enough light reflected from the surface (20, 21). Second, due to the posture changes in different fractions, the surface is prone to deformation. Third, the surface is symmetrical along the VRT direction, which can affect the positioning accuracy in LNG and VRT direction (32). Different from Sentinel and Catalyst, S-M_OPS enhance the reflective light by coating positioning sphere’s surface with reflective material. Second, S-M_OPS used thermoplastic mask to immobilize the patient to maintain a relatively invariant posture. Third, the distribution of positioning spheres is asymmetric. Therefore, S-M_OPS-guided setup accuracy and stability are higher. For ExacTrac, it needs X-ray imaging to assist with setup. However, X-ray imaging not only brings additional radiation, but also takes a lot of time. According to Linthout N's clinical trial report, the average time of ExacTrac-guided setup was 191s (33), which was much greater than time consumption of S-M_OPS-guided setup (7.47s). In addition, in terms of the complexity of the operation, ExacTrac needs X-ray imaging. Sentinel and Catalyst need to adjust parameters (gain and integration time) (20, 21), but S-M_OPS doesn’t need additional operation, which bring convenience to radiotherapists.

**Table 2:** Comparisons among S-M_OPS-guided setup errors, Sentinel-guided setup errors, Catalyst-guided setup errors and ExacTrac-guided setup errors.

		Head and neck/mm	Thorax/mm	Pelvis/mm	Overall/mm
LAT	S-M_OPS	0.1 ± 2.1	-0.1 ± 1.9	-0.6 ± 1.6	-0.2 ± 1.9
	Sentinel	0.9 ± 1.8	1.2 ± 3.6	-2.5 ± 4.1	-1.0 ± 3.6
	Catalyst	0.3 ± 2.4	0.7 ± 3.3	1.2 ± 2.5	-0.7 ± 2.8
	ExacTrac	4.1 ± 2.7	0.1 ± 1.8	-0.6 ± 2.7	N/A
VRT	S-M_OPS	1.0 ± 2.1	1.1 ± 1.8	1.0 ± 1.8	1.0 ± 1.9
	Sentinel	-2.7 ± 3.8	0.8 ± 5.1	-4.6 ± 7.3	1.0 ± 6.3
	Catalyst	-3.7 ± 3.4	-0.7 ± 3.8	0.2 ± 3.7	-1.3 ± 4.0
	ExacTrac	1.2 ± 0.5	0.2 ± 2.4	-0.3 ± 2.3	N/A
LNG	S-M_OPS	-0.4 ± 1.7	0.2 ± 3.2	-0.4 ± 2.4	-0.3 ± 2.3
	Sentinel	-0.8 ± 3.6	0.8 ± 4.3	-5.1 ± 7.4	-1.8 ± 5.9
	Catalyst	-0.2 ± 3.4	2.4 ± 3.2	1.8 ± 3.7	1.5 ± 3.6
	ExacTrac	1.1 ± 0.7	-0.6 ± 1.8	1.6 ± 3.5	N/A

Values are shown in mean ± SD with best shown with shading. NA, not applicable.

## 5 Conclusion

In conclusion, the setup accuracy and stability of S-M_OPS are significantly higher than those of laser line, Sentinel, Catalyst and ExacTrac. What’s more, S-M_OPS has the comparable setup accuracy to CBCT and the shorter setup time, which is especially suitable for countries with insufficient radiotherapy resources.

## Data availability statement

The raw data supporting the conclusions of this article will be made available by the authors, without undue reservation.

## Ethics statement

The studies involving human participants were reviewed and approved by Medical Technology Access Management Committee, Medical Department, Nantong Tumor Hospital. The patients/participants provided their written informed consent to participate in this study. Written informed consent was obtained from the individual(s) for the publication of any potentially identifiable images or data included in this article.

## Author contributions

YZ and YG contributed to the concept of the study. YZ, HZ, YG, WS, YC and XH reviewed the manuscript. YZ, YG designed the study and did the literature search. YZ, KC, CW, YG, GS, JZ, JC and JJ collected the data. YZ, KC, CW, YG, GS, JZ, JC and JJ contributed to the data analysis and data interpretation. YZ and YG drafted the manuscript. YZ, HZ, YG, YC and XH revised the manuscript. YZ and YG have verified the data and had final decision to submit for publication. All authors contributed to the article and approved the submitted version.

## Funding

This study was supported by the Jiangsu Provincial Social Development Key R&D Program (BE2020685), the National Natural Science Foundation of China (81973872), the Natural Science Foundation of Jiangsu Province (BK20191250) and the Nanjing Medical University Science and Technology Development Fund (NMUB2020271).

## Conflict of interest

The authors declare that the research was conducted in the absence of any commercial or financial relationships that could be construed as a potential conflict of interest.

## Publisher’s note

All claims expressed in this article are solely those of the authors and do not necessarily represent those of their affiliated organizations, or those of the publisher, the editors and the reviewers. Any product that may be evaluated in this article, or claim that may be made by its manufacturer, is not guaranteed or endorsed by the publisher.

## Appendix

Jiangsu Provincial Hospital of Chinese Medicine

Nantong Tumor Hospital

Subei People's Hospital of Jiangsu Province

Suzhou Municipal Hospital

Huai'an Second People's Hospital

YYixing Tumor Hospital

Baoying People's Hospital

Yangzhou First People's Hospital

Zhejiang Cancer Hospital

Xinghua City People's Hospital

The People's Hospital of Yizheng

Jiangsu Corps Hospital of Armed Police Forces
